# Mineral coated microparticles doped with fluoride and complexed with mRNA prolong transfection in fracture healing

**DOI:** 10.3389/fbioe.2023.1295313

**Published:** 2024-01-09

**Authors:** Anna Laura Nelson, Gianluca Fontana, Laura Chubb, Josh Choe, Katherine Williams, Dan Regan, Johnny Huard, William Murphy, Nicole Ehrhart, Chelsea Bahney

**Affiliations:** ^1^ Center for Regenerative and Personalized Medicine, Steadman Philippon Research Institute (SPRI), Vail, CO, United States; ^2^ School of Biomedical Engineering, Colorado State University, Fort Collins, CO, United States; ^3^ Department of Orthopedics and Rehabilitation, University of Wisconsin-Madison, Madison, WI, United States; ^4^ Department of Clinical Sciences, Colorado State University, Fort Collins, CO, United States; ^5^ Department of Microbiology, Colorado State University, Fort Collins, CO, United States; ^6^ Department of Biomedical Engineering, University of Wisconsin-Madison, Madison, WI, United States; ^7^ Orthopaedic Trauma Institute, University of California, San Francisco, CA, United States

**Keywords:** bone regeneration, mRNA delivery, biomimetic, transfection, fluoride

## Abstract

**Introduction:** Impaired fracture healing, specifically non-union, has been found to occur up to 14% in tibial shaft fractures. The current standard of care to treat non-union often requires additional surgeries which can result in long recovery times. Injectable-based therapies to accelerate fracture healing have the potential to mitigate the need for additional surgeries. Gene therapies have recently undergone significant advancements due to developments in nanotechnology, which improve mRNA stability while reducing immunogenicity.

**Methods:** In this study, we tested the efficacy of mineral coated microparticles (MCM) and fluoride-doped MCM (FMCM) to effectively deliver firefly luciferase (FLuc) mRNA lipoplexes (LPX) to the fracture site. Here, adult mice underwent a tibia fracture and stabilization method and all treatments were locally injected into the fracture. Level of osteogenesis and amount of bone formation were assessed using gene expression and histomorphometry respectively. Localized and systemic inflammation were measured through gene expression, histopathology scoring and measuring C-reactive protein (CRP) in the serum. Lastly, daily IVIS images were taken to track and measure transfection over time.

**Results:** MCM-LPX-FLuc and FMCM-LPX-FLuc were not found to cause any cytotoxic effects when tested *in vitro*. When measuring the osteogenic potential of each mineral composition, FMCM-LPX-FLuc trended higher in osteogenic markers through qRT-PCR than the other groups tested in a murine fracture and stabilization model. Despite FMCM-LPX-FLuc showing slightly elevated *il-1β* and *il-4* levels in the fracture callus, inflammation scoring of the fracture callus did not result in any differences. Additionally, an acute systemic inflammatory response was not observed in any of the samples tested. The concentration of MCM-LPX-FLuc and FMCM-LPX-FLuc that was used in the murine fracture model did not stimulate bone when analyzed through stereological principles. Transfection efficacy and kinetics of delivery platforms revealed that FMCM-LPX-FLuc prolongs the luciferase signal both *in vitro* and *in vivo*.

**Discussion:** These data together reveal that FMCM-LPX-FLuc could serve as a promising mRNA delivery platform for fracture healing applications.

## Introduction

Fractures continue to be one of the most frequent type of hospitalized traumas with reports of about 32.7 million new cases of lower leg fractures globally in 2019 (Ekegren et al., 2018; Cauley, 2021). Additionally, tibial shaft fractures account for about 36.7% of all long-bone fractures in adults and are frequently associated with high-energy trauma events, like falls and transport accidents (Weiss et al., 2008; Papakostidis et al., 2011; Ekegren et al., 2018). Open tibial fractures are often associated with higher rates of complications, like non-union or delayed union (Gaebler et al., 2001; Papakostidis et al., 2011). In fact, reports of non-union have been found to occur up to 14% within tibial shaft fractures in a large study over the course of 2 years (Bisignano et al., 2021). Non-union is typically treated through repeated surgeries resulting in long recovery times leading to increased frailty, depression, loss of independence, and, in some cases, a downward spiral that ends in death (Colón-Emeric and Saag, 2006; Gruber et al., 2006). Bone morphogenetic protein-2 (BMP2) is a well-studied osteoanabolic that accelerates fracture healing in preclinical models, yet only has FDA approval in lumbar fusion and acute open tibial fractures (9–13) Recombinant human proteins have short half-lives and require supraphysiologic concentrations to achieve the desired biologic effect (12) In order to sustain protein release, typically proteins are delivered on an absorbable collagen sponge, necessitating surgical implantation (13–15) Despite this controlled release system, adverse side effects have continued to be reported with BMP-2 including abnormal inflammatory responses, poor bone quality, heterotopic ossification, and osteolysis (14, 16–18) Given the few approved therapeutics to accelerate fracture healing, and the limitations of those available, there is a clinical need to develop novel, and injectable-based therapies to accelerate fracture healing and minimize the incidence of delayed and non-union fractures.

Over the past few decades, the demand for orthopaedic-based technologies has followed the substantial growth in orthopaedic surgeries being performed each year (Su et al., 2022). Of these emerging orthopaedic technologies, gene therapy has undergone significant advancements due to developments in delivery carriers and their capabilities to maximize gene stability, minimize off-target effects, and reduce immunogenicity (Mockey et al., 2006; Zohra et al., 2007; Mukherjee and Thrasher, 2013; Ramunas et al., 2015; Sultana et al., 2017; Shirley et al., 2020; Watson-Levings et al., 2022). Specifically, the considerable success of the SARS-CoV-2 vaccine has catalyzed research efforts in developing mRNA-based technologies as safety and efficacy have been established with mRNA therapeutics (Altawalah, 2021; Ward et al., 2022; Watson-Levings et al., 2022). mRNA-based therapy in orthopedics remains a nascent field, and specifically for skeletal tissues as these are prone to clinical challenges such as multifactorial pathologies and complex healing mechanisms (Evans et al., 2021; Watson-Levings et al., 2022). Recent work pioneering mRNA therapies to promote bone regeneration through coding for BMP2/9 has shown considerable promise, yet all studies use a biomimetic scaffold requiring surgical implantation (Balmayor et al., 2016; Khorsand et al., 2017; De La Vega et al., 2022). We propose that the ideal mRNA delivery platform to promote fracture repair should include a localized and injectable therapeutic, mitigating the need for additional surgeries in impaired fracture healing cases.

Effective mRNA delivery to cells requires the use of delivery carriers to protect the nucleic acids from degradation from nucleases (Kowalski et al., 2019; Nitika et al., 2022). Currently liposomal complexes are the most commonly utilized delivery vehicles for mRNA based therapeutics, including lipid nanoparticles and liposomes (Kowalski et al., 2019; Nitika et al., 2022). However, lipoplexes can induce immunogenic reactions and are rapidly cleared by the mononuclear phagocytic system (MPS) through the kidney, liver, and spleen (Kowalski et al., 2019). Alternative strategies have been employed to mitigate these limitations and more effectively transport genes important in bone repair. β-tricalcium phosphate mineral coated microparticles (Alluri et al., 2019; Fontana et al., 2019; Kang et al., 2021) (MCM) which consist of a bioresorbable core material coated with calcium phosphate resulting in microparticles 5–8 µm in diameter with a thin, plate-like mineral coating on their surface (Choi and Murphy, 2010; Yu and Murphy, 2014; Fontana et al., 2019). Recently published studies demonstrate that MCMs can enhance mRNA transfection when combined with lipoplexes and mitigate cytotoxicity resulting from high *in vitro* mRNA concentrations (Dang et al., 2016; Khalil et al., 2017; Fontana et al., 2019).

In this study, we have developed and tested an MCM-mRNA delivery platform for its capacity to effectively deliver a reporter mRNA in a fracture healing setting. The biomimetic mineral coating localizes the delivery of lipoplexes to cells and has been found to prolong the biological function of various biomolecules being delivered *in vivo* testing various applications, such as for spinal cord injuries (Khalil et al., 2017; Fontana et al., 2019; Khalil et al., 2020; Khalil et al., 2022). The use of MCMs to localize lipoplexed mRNA and improve cell internalization of mRNA at the fracture callus addresses several limitations, including the immunogenicity associated with liposomal carriers. Here we show that mRNA transfection is enhanced, prolonged, and does not interfere with fracture healing when using MCMs as a transfection platform in a murine tibial fracture healing model.

## Methods and materials

### Synthesis of mineral coated microparticles and fluoride-doped MCM and microparticle characterization

Mineral coated microparticles were made from β-tricalcium phosphate (β-TCP) 2 µm powder (Cambioceramics, Amsterdam, Netherlands) as the microparticle core and incubated in modified simulated body fluid (mSBF) at 1 mg/mL for 7 days. The mSBF was prepared by using 141 mM NaCl, 4 mM KCl, 0.5 mM MgSO4, 1 mM MgCl2, 4.2 mM NaHCO3, 20 mM HEPES, 5 mM CaCl2, and 2 mM KH2PO4. For Fluoride-doped MCM (FMCM) we introduced 1 mM NaF to the standard mSBF. All microparticles were maintained in suspension at 37°C with rotation. The microparticles were centrifuged at 2,000 x g for 5 min every 24 h over 7 days. The supernatant was discarded and replenished with fresh mSBF. Post 7 days, the MCMs were rinsed three times using 50 mL deionized water and filtered using a 40 µm pore cell strainer. Finally, the MCMs were concentrated and subjected them to lyophilization for 48 h (Fontana et al., 2019). Dry MCMs were placed on carbon tape and sputter coated with 10 nm of platinum (ACE600, Leica Microsystems, Morrisville, NC, United States). MCMs were imaged at 5.0 KV using a Gemini 450 scanning electron microscope (Zeiss, Jena, Germany).

### Cell culture maintenance and cytotoxicity experiments

The chondrogenic cell line (ATDC5, Sigma Aldrich, Cat # 99072806) was used for all *in vitro* studies. Cells were used at passage 5 and under for all experiments. ATDC5 cells were maintained using media DMEM/F12 (Thermo Fisher, Cat# 11320033), 5% fetal bovine serum (FBS, Thermo Fisher, Cat# 16000044) and 1% penicillin/streptomycin (P/S, Thermo Fisher, Cat# 15140122). For all *in vitro* experiments, ATDC5 cells were plated at 20,000 cells/well in 12 well plates. Prior to transfection experiments, chondrocytes underwent serum starvation using basal media OPTI-MEM (Thermo Fisher, Cat# 31985070) 0.5% FBS and 1% P/S for 24 h. All transfection protocols were executed using serum-free media, OPTI-MEM supplemented with 1% P/S. The cytotoxic effects were assessed using PrestoBlue™ Cell Viability Reagent (Thermo Fisher, Cat# A13261) according to the manufacturer’s protocol. To quantify the number of metabolically active cells, a standard curve of ATDC5 cells was created and the absorbance values were related back to the number of cells for quantitation. The plate was read at 570 nm with a reference wavelength at 600 nm using a plate reader (TECAN infinite M200 Pro).

### Mineral coated microparticles and fluoride-doped MCM formulations with lipoplexes

MCM and FMCM were used as a platform to deliver lipid vesicles encapsulating mRNA to the chondrocytes *in vitro* and to the site of the fracture callus *in vivo*. To determine transfection protocols and concentrations for effective delivery of mRNA, firefly luciferase (FLuc) mRNA was used as a reporter transcript (TriLink Biotech, Cat# L-7202) for all experiments. Lipoplexes (LPX), liposomes complexed with mRNA, were generated using Lipofectamine™ MessengerMAX (ThermoFisher, Cat# LMRNA001) and firefly luciferase mRNA according to manufacturer’s protocol. To combine the LPXs with MCM and FMCM, the LPX solution was added to MCM or FMCM in OPTI-MEM for *in vitro* studies or sterile filtered 1X phosphate buffer solution (PBS, Thermo Fisher, Cat # 10010023) for *in vivo* studies, with shaking for 30 min at RT. All *in vitro* experiments used 0.25 μg of firefly luciferase mRNA/well, 2 μL of Lipofectamine™/well, and 100 μg of MCM or FMCM per well. *In vivo* analysis of transfection reagents used firefly luciferase mRNA at a concentration of 10 µg/mouse, Lipofectamine™ at 15 µL/mouse, and MCM/FMCM at 100 µg/mouse. Firefly luciferase expression in MCM-Lipofectamine™-FLuc mRNA platform (MCM-LPX-FLuc) and FMCM-Lipofectamine™-FLuc mRNA (FMCM-LPX-FLuc) was compared to the luciferase expression from Lipofectamine™-FLuc mRNA (LPX-FLuc).

### Tibial fracture and stabilization procedure in mice

*In vivo* experiments were approved by and conducted in compliance with the Institutional Animal Care and Use Committee at Colorado State University (CSU). Mid-shaft tibial fractures were created in adult (12–14 weeks), male C57BL/6J mice (Charles River # 027). All animals received a pre-surgical analgesic, Buprenorphine SR (ZooPharm), at a dose of 0.6–1.0 mg/kg. Mice were placed under general anesthesia using inhaled 1%–5% isoflurane to effect. Left hind limbs were shaved and sterilely prepared using 70% alcohol wipes and Chlorhexidine surgical scrub solution, repeated for a total of 3 times. Lubrication was provided for the eyes of each mouse using artificial tears ointment (Bausch and Lomb) and mice were then transferred to a heated operating table. Using aseptic technique, an incision was made along the tibia, and a hole was made at the top of the tibial plateau using a 23-gauge needle. An intermedullary pin (sterilized insect pin) was inserted through the hole made from the tibial plateau through the tibial cavity and into the distal tibia. Two to three small holes were created in the mid-shaft of the tibia using a Dremel and pressure was applied to both proximal and distal ends to fracture the tibia (Hu et al., 2017; Wong et al., 2020). The surgical incision was then sutured using 5–0 Biosyn Sutures (Covidien, Cat #5687) and one surgical skin staple was applied over the skin incision to protect against chewing. A local anesthetic, 0.25% Bupivicaine Hydrochloride (NovaPlus, cat # RL-7562), was applied topically after the initial staple was placed. Mice were then allowed to recover individually in a heated cage before being transferred back to their original cage. Mice were socially housed throughout the in-life period, and permitted free ambulation. Mice were closely monitored for pain and signs of infections for 72 h following the surgery. All treatments were suspended in 25 μL sterile PBS and vortexed before injecting. All experimental and control groups were injected on day 6 post-fracture into the fracture callus using a Hamilton syringe 1800 series (Sigma Aldrich, Cat # 21397). Localized treatments consisted of experimental groups (MCM-LPX and FMCM-LPX), negative and positive control groups (PBS and LPX respectively). Serum was collected 2 days following treatment via tail vein blood draw and lastly at time of harvest using a cardiac puncture. Animals were sacrificed according to approved euthanasia protocols after 2 or 8 days following localized treatments. At euthanasia, serum, fractured tibia, kidneys, liver spleen and lung were harvested for later analysis. Harvested tibias were either fixed in 4% paraformaldehyde for histological analysis or flash-frozen in liquid nitrogen for RNA analysis. Tissues and serum were flash frozen by immediately placing in liquid nitrogen and stored at −80°C.

### *In vitro* and *in vivo* RNA isolation and qRT-PCR

RNA was isolated using TRIzol™ Reagent (ThermoFisher, Cat# 15596026) according to the manufacturer’s guidelines. To isolate RNA from all frozen tissues, the tissues were minced and placed in 1 mL of TRIzol Reagent and were homogenized using IKA Tissue Homogenizer (IKA, Cat# 0003737001) using a speed of 5 for 3 min, or at least until all large chunks were broken down. Specifically, the fracture callus was isolated from the surrounding muscle and bone tissues prior to homogenization. RNA was then quantified by reading the absorbance values at 260 and 280 nm. 1 μg of RNA was then synthesized into cDNA using qScript cDNA SuperMix (QuantaBio, Cat# 95048-025). Primers were designed and are listed below. Quality measurements were used to confirm primer specificity and appropriate reaction temperatures by running PCR using DreamTaq Green PCR Master Mix (2X) (ThermoFisher, Cat# K1081) using a 3-step method with denaturation at 95°C for 30 s, annealing at 60°C for 30 and extension at 72°C for 1 min. The bands were analyzed on a 1.5% agarose gel with 2 µL GelStar Nucleic Acid Stain (Lonza, Cat # 50535) run at 150V for 25 min. Quantitative real-time PCR was run using SYBR Green Master Mix (Bio-Rad, Cat#1725270) to detect the amplified DNA. qRT-PCR was performed on a StepOnePlus™ instrument (Applied Biosystems). To analyze the output Ct values, the reference housekeeping gene (β-2-modulin) was used to determine ∆Ct values. The value of 2-(∆∆Ct) was calculated for all graphs and to determine statistical significance. All 2(−ΔΔCt) values less than 1.0, were calculated as −1/(2(−ΔΔCt)) as followed and described in Schmittgen and Livak, 2008.

### *In vitro* and *in vivo* immunogenicity, osteostimulatory and transfection efficacy testing

Analysis of transfection formulations for all *in vitro* studies was measured after collecting RNA at 3, 6, 24, and 48 h after treatments and for *in vivo* studies, RNA was collected 8 days after treatments. To measure *in vitro* and *in vivo* immune responses, qRT-PCR was used testing for pro-inflammatory marker, *interleukin-1β (il-1β),* and anti-inflammatory marker, *interleukin-4 (il-4).* Local inflammatory response was measured using qRT-PCR and histopathology while systemic inflammation was tested using a C-Reactive Protein (CRP) ELISA kit (R&D Systems, Cat# MCRP00) according to manufacturer’s instructions on the serum collected at day 2 and 8 post-treatment (Morioka et al., 2019). Osteostimulatory characterization was determined by using qRT-PCR for osteogenic markers *osterix* (*osx*), *osteocalcin (ocn*), *collagen 10a1* (*colX*) and downstream canonical Wnt markers, *axis inhibition protein 2* (*axin2)* and *runt-related transcription factor* (*runx2)*. Firefly luciferase primers were used to measure luciferase expression to determine magnitude of luciferase signal and kinetics of transfection.

### Histopathological scoring

Tibias of treated mice were collected 48 h post treatment, immersion fixed in 4% paraformaldehyde, and decalcified using 19% ethylenediaminetetraacetic acid (EDTA). Formalin-fixed tibial samples were embedded in paraffin, sectioned at 8 μm thickness, and mounted onto glass slides. Two serial sections of each sample were cut and stained with hematoxylin and eosin using routine methods. Semiquantitative histopathologic analysis was performed by a board-certified veterinary pathologist (D.P.R.) who was blinded to the treatment arm. For analysis, slides were scanned on low magnification and the fracture callus region-of-interest identified and histological scoring performed based on the average cellular response across this callus region-of-interest. Semi-quantitative scoring was performed using a modification of ISO10993-6 Annex E: Biological evaluation of medical devices–Part 6: Tests for local effects after implantation (Supplementary Table S1). Cellular and host tissue response parameters assessed included the inflammatory cell types of polymorphonuclear cells (PMNs), lymphocytes (lymphs), plasma cells, macrophages, and giant cells, as well as necrosis, edema, hemorrhage, neovascularization and fibrosis. Responses were scored based on a 0–4 scale where 0 represents “none,” 1 represents “rare/minimal,” 2 represents “mild,” 3 represents “heavy infiltrate/moderate” and 4 represents “packed/severe.”

### Real-time bioluminescent imaging

IVIS imaging was performed daily to quantitatively assess the magnitude and duration of luciferase expression from the time of delivery of reporter mRNA until signal dissipated. All mice were anesthetized using isoflurane and injected with 100 µL of firefly luciferase substrate, D-luciferin, subcutaneously at 10 mg/mL in PBS. Continued isoflurane was maintained at 1%–5% and mice were imaged 5 min following subcutaneous injection of D-luciferin. Bioluminescence was acquired using the “Auto” setting and regions of interest were measured for bioluminescence using LivingImage software (PerkinElmer, version). Percent retention was calculated using the following equation for each animal:

$$\%\text{Retention}=100\%‐\left[\left(\left(A‐B_{x}\right)/A\right)\;x\;100\%\right]$$

where A = IVIS output from day 1, and B_x_ = IVIS output from day x.

### Histological and histomorphometric analysis

Bilateral tibias were decalcified for 14–18 days using 19% EDTA, pH 7.4. The tissues were then processed in increased ethanol solutions (50%, 70%, 95%, and 100%), processed in xylene (x2) and then placed in paraffin for 1.5 h each. Tissues were then paraffin embedded and sectioned at 8 µm. Immunohistochemistry (IHC) was performed to identify transfected cells within the fracture callus using previously published methods (Bahney et al., 2014; Hu et al., 2017). Antigen labeling was performed using a primary anti-luciferase (Novus Biologicals, Cat# NM600-307) antibody. Negative control mouse IgG monoclonal antibody (ThermoFisher, Cat# 10400C) was used in replace of firefly luciferase antibody to determine non-specific stain. Cell morphology was used as the primary tool to identify specific immunopositive cell types, using Hall Brundt’s Quadruple (HBQ) Stain on adjacent slides (Hall, 1986). The following kits were used for IHC detection: Mouse on Mouse Immunodetection Kit (Vector Laboratories, Cat# BMK2202), VectaStain Elite ABC-HRP Kit (Vector Laboratories, Cat# PK-6200) and DAB Substrate Kit, Peroxidase (HRP) (Vector Laboratories, Cat# SK-4100). All kits were used according to manufacturer’s protocol. Sections were counterstained using Hematoxylin, dehydrated and mounted in a xylene-based mounting medium.

To perform quantitative histomorphometry, serial sections were obtained using the first section beginning in the fracture callus and every 10th section afterwards. Standard histomorphometry principles were used as previously described and quantification of the fracture callus components including bone and cartilage tissues were determined (Hu et al., 2017; Rivera et al., 2021). HBQ stain was used to identify the dense collagenous fibrils of bone, stained red by direct red, and proteoglycans in the cartilage matrix, stained by alcian blue. Quantification of callus composition was determined using the Trainable Weka Segmentation2 add-on in Fiji ImageJ (version 1.54f; NIH, Maryland, United States) (Malhan et al., 2018). Volume of specific tissue types was determined by summing the individual compositions relative to the whole fracture callus compositions. Tissues were imaged using a Nikon Eclipse Ti microscope. Additionally, photoshop (version 24.7.0, Adobe) was used to isolate fractured tissue from the adjacent muscle and skin tissues.

### Statistical design

Animal sample size was determined *a priori* using the mean and standard deviation from our preliminary data, where a power analysis was conducted using G*Power to determine that 5 mice/group/time are required for IVIS imaging to achieve a power level >80% with an effect size d = 1.5 and a significance level of 5%. Statistical analysis was performed using Graph Pad Prism 8. Data were plotted so that each sample represented a single dot on each graph. The bar indicates the mean with error bars representing standard deviation. Statistical difference was determined by ANOVAs and all *post hoc* comparison performed using Tukey’s HSD test.

## Results

### Mineral coated microparticles and fluoride-doped MCM characterization and metabolic testing

**FIGURE 1:**
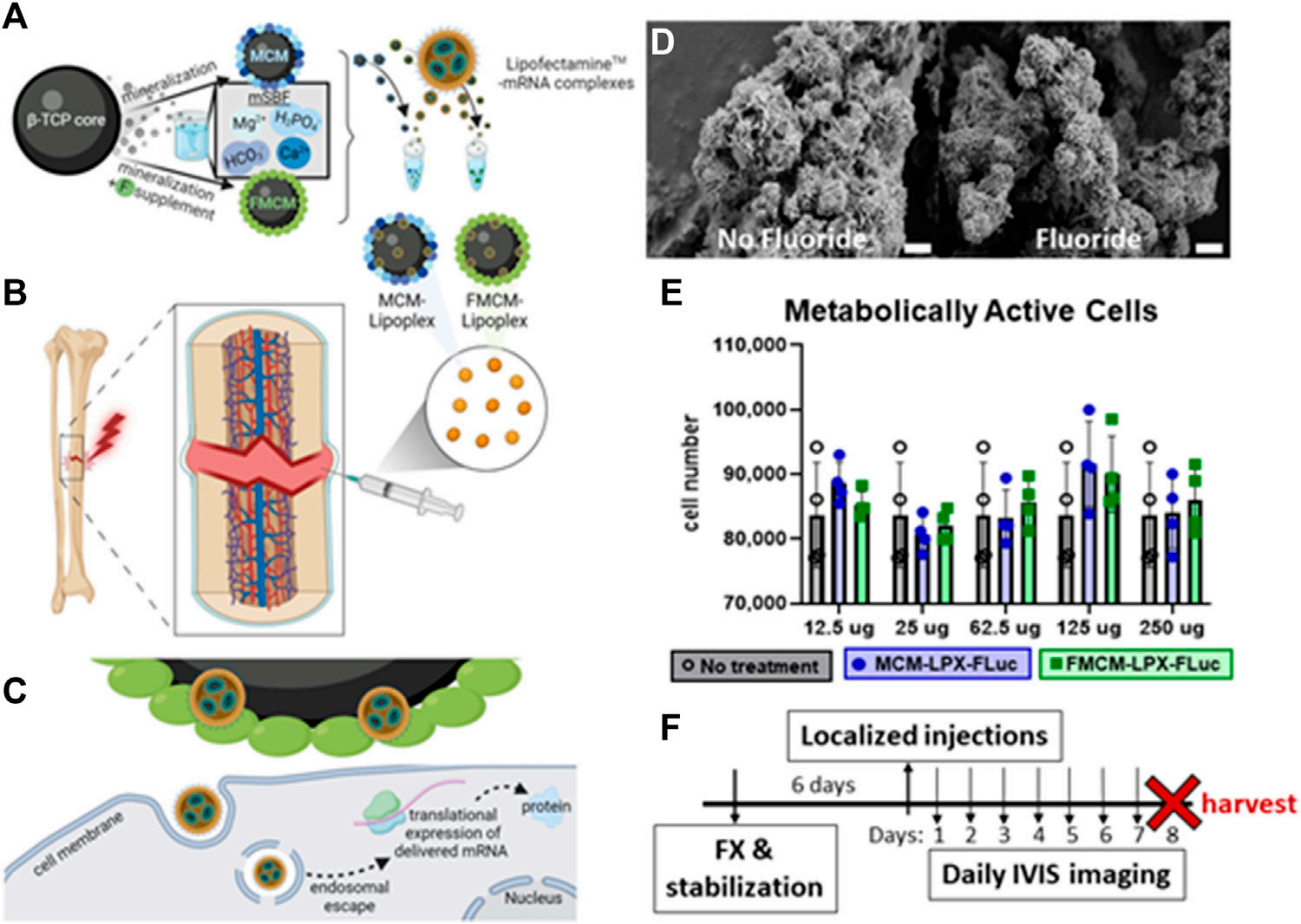
Schematic of MCM and FMCM synthesis, delivery, cell interactions and characterization of microparticles. Schematic of MCM and FMCM synthesis and lipoplex interaction **(A)**. Delivery of test article and controls were localized at the site of the fracture callus **(B)**, where MCM-LPX-FLuc and FMCM-LPX-FLuc are not endocytosed by the cells **(C)**. SEM imaging of MCM (left) and FMCM (right) **(D)**. Scale bars represent 2 μm. Metabolic activity was measured to determine cytotoxicity of MCM-LPX-FLuc and FMCM-LPX-FLuc **(E)**. Timeline of *in vivo* studies, where injections were performed 6 days after fracture and stabilization in a murine model **(F)**. Schematic created with Biorender.com.

A schematic depicting the generation of MCM-LPX-FLuc and FMCM-LPX-FLuc can be viewed in Figure 1A, beginning with the synthesis of MCM and FMCM from a β-TCP core, and finally complexed with LPXs. All injections were performed locally to the site of the fracture callus (Figure 1B), where the MCM-LPX-FLuc and FMCM-LPX-FLuc are not endocytosed by the cell, but act as a carrier for the LPX-FLuc, which undergo endocytosis. mRNA is then expressed as protein using the cell’s internal machinery (Figure 1C). MCM and FMCM were characterized using scanning electron microscopy (SEM) to visualize the outer morphology of the microparticles (Figure 1D). MCM were found to have a plate-like outer coating where FMCM had a more needle-like structure. The cytotoxic effects of the microparticles were evaluated and were found to have no significant *in vitro* chondrocyte cytotoxicity at any dose tested (12.5 μg–250 μg) (Figure 1E). Based on this data, 100 μg of MCM or FMCM was chosen to use in all *in vitro* and *in vivo* experiments, as this concentration did not promote cytotoxic effects when *in vitro* and additionally, this concentration has previously shown success in a murine spinal cord injury model (Khalil et al., 2022). All animal experiments were conducted according to the timeline outlined in Figure 1F, where injections of test article or control were performed 6 days following the fracture.

### Mineral coatings stimulate osteogenesis

**FIGURE 2:**
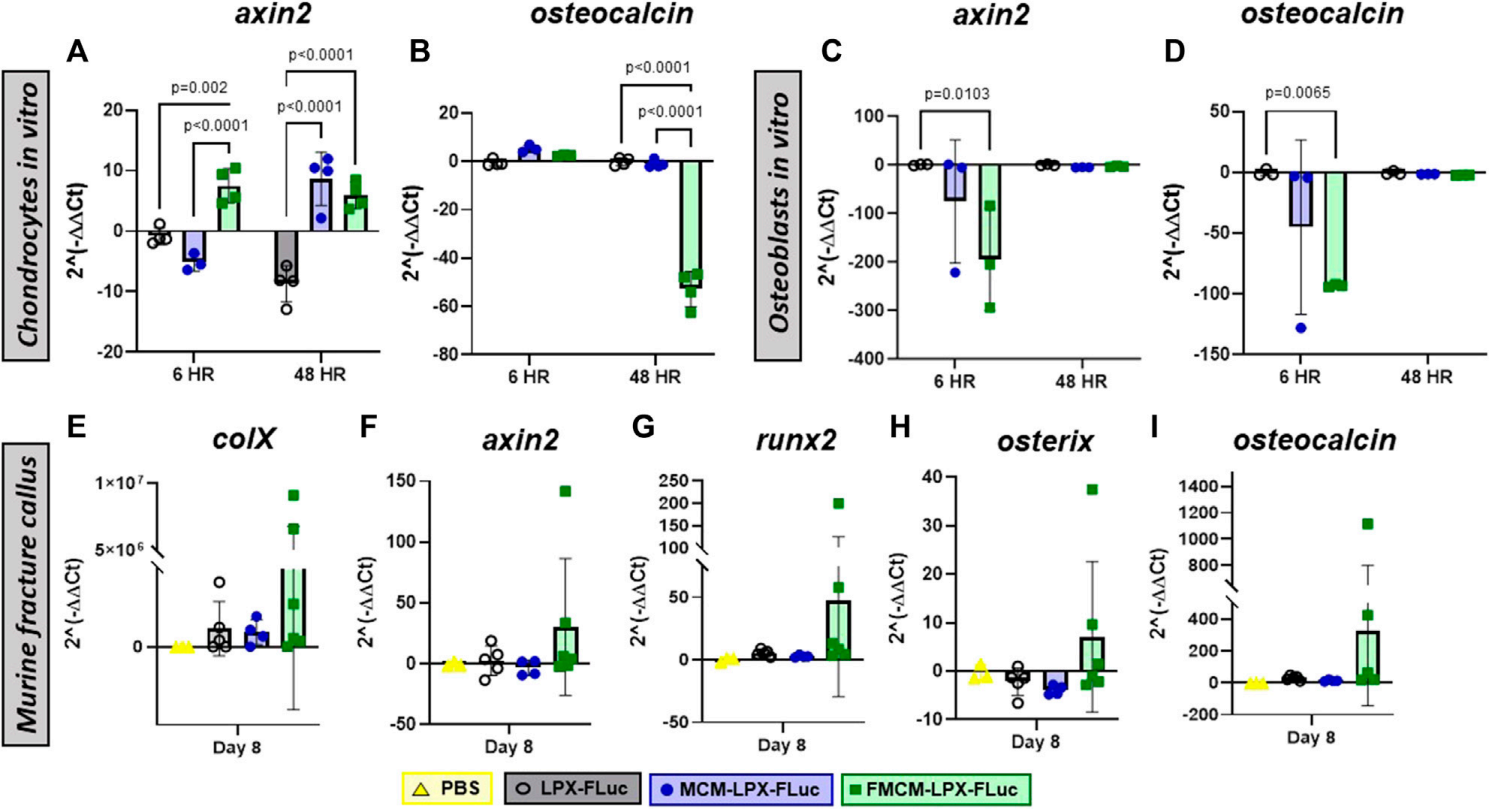
Osteogenic potential was tested through qRT-PCR in chondrocytes **(A, B)** and osteoblasts **(C, D)** *in vitro*, and within the fracture callus in a murine model *in vivo* **(E–I)**.

As it has previously been shown that mineral coatings have some osteogenic characteristics *in vitro* using various cell types, such as progenitor osteoblasts and mesenchymal and embryonic stem cells, we tested whether there was any osteostimulatory effect in chondrocytes and osteoblasts (Murphy et al., 2004; Chou et al., 2005; Choi and Murphy, 2010; Choi et al., 2013; Dang et al., 2016; Wang et al., 2016). When probing for osteogenic markers *in vitro*, MCM-LPX-FLuc was found to have more *ocn* expression at every time point tested in chondrocytes as compared to FMCM-LPX-FLuc (Figure 2B), yet FMCM-LPX-FLuc and MCM-LPX-FLuc both exhibited significant *axin2* expression at later time points in chondrocytes (Figure 2A). Osteogenic markers, *axin2* and *ocn*, were significantly downregulated in osteoblasts following treatment with FMCM-LPX-FLuc after 6 h, yet this significance dissipated by 48 h (Figures 2C,D). Within the tibia fracture callus, both earlier osteogenic genes, *collagen 10a1*, *axin2,* and *runx2,* and late osteogenic genes, *osterix* and *osteocalcin,* were tested for a more robust analysis of MCM-LPX-FLuc and FMCM-LPX-FLuc osteogenic potential. While no statistical significance was found *in vivo*, a trend of higher expression of osteogenic genes was observed with the FMCM-LPX-FLuc group (Figures 2E–I).

### MCM-LPX-FLuc and FMCM-LPX-FLuc do not stimulate an inflammatory response

**FIGURE 3:**
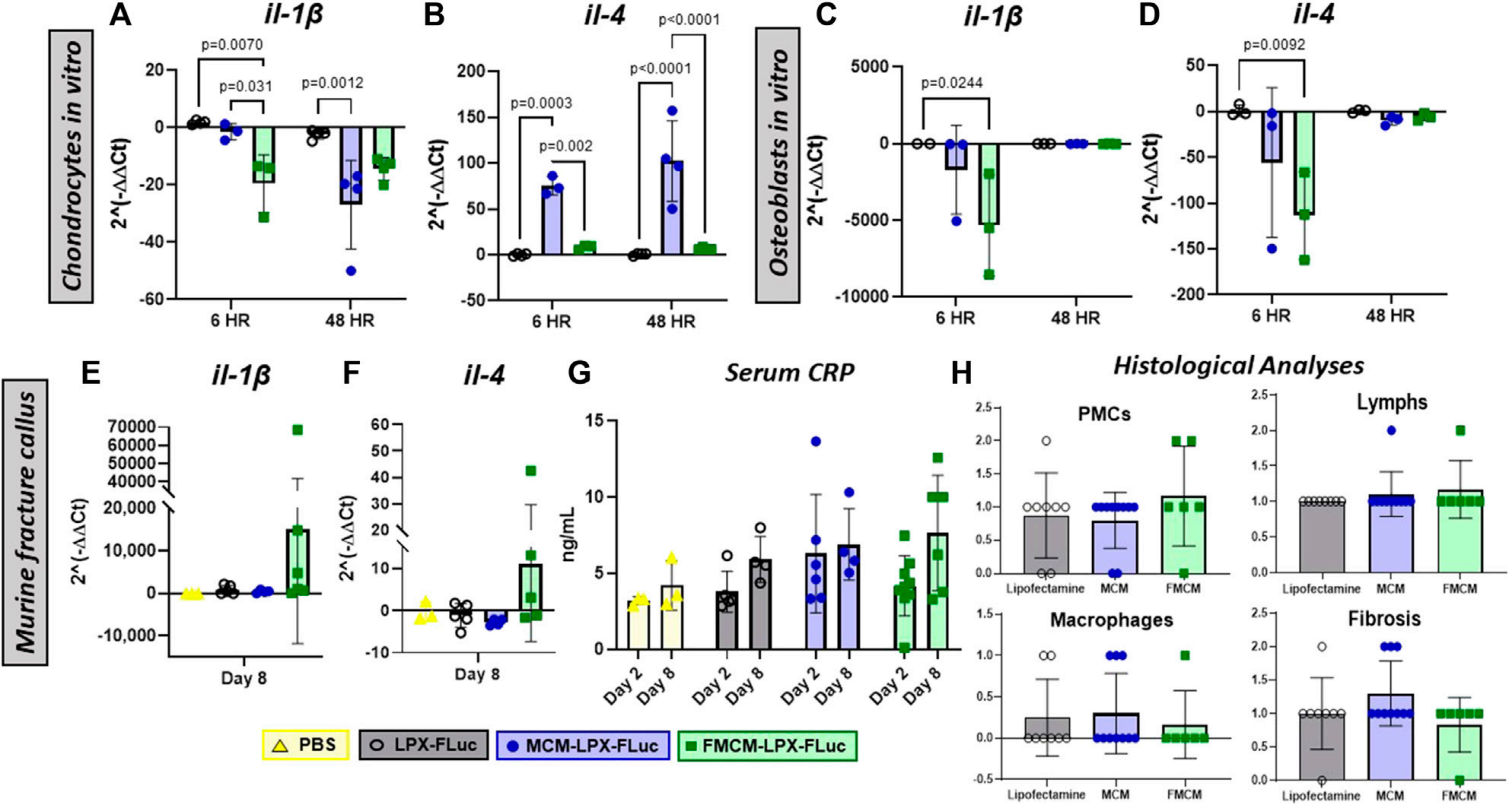
Inflammatory responses were measured by using qRT-PCR for pro-inflammatory marker, il-1β, **(A,C and E)** and anti-inflammatory marker, il-4, **(B,D and F)** both *in vitro* and within the fracture callus to measure localized inflammatory response following treatments. Systemic inflammation was tested by quantifying C-reactive protein within the serum both 2 and 8 days after local injections **(G)**. Additionally, H&E sections of the fracture callus were scored by a pathologist for peripheral mononuclear cells, lymphocytes, macrophages and fibrosis, 2 days after treatments **(H)**.

We aimed to test localized and systemic inflammatory responses following treatments with MCM-LPX-FLuc and FMCM-LPX-FLuc. Overall, both FMCM-LPX-FLuc and MCM-LPX-FLuc were found to have less *il-1β* expression at all time points in both chondrocytes and osteoblasts. Specifically, FMCM-LPX-FLuc was found to have significantly less *il-1β* at earlier time points tested in both chondrocytes and osteoblasts as compared to the LPX-FLuc group alone (Figures 3A,C). The MCM-LPX-FLuc was found to have significantly less *il-1β* when tested in chondrocytes at 48 h compared to the LPX-FLuc group (Figure 3A). Additionally, MCM-LPX-FLuc was found to have significantly more *il-4* in chondrocytes at all time points as compared to both LPX-FLuc and FMCM-LPX-FLuc groups (Figure 3B), yet no significant differences with MCM-LPX-FLuc group were found when tested in osteoblasts (Figure 3D). FMCM-LPX-FLuc group was found to have significantly less *il-4* expression at 6 h in osteoblasts as compared to LPX-FLuc, yet this significance dissipated by 48 h (Figure 3D).

While no significance was found, modulation of inflammatory markers *in vivo* showed FMCM-LPX-FLuc to trend higher in *il-1β* and *il-4* as compared to both MCM-LPX-FLuc and LPX-FLuc groups (Figures 3E,F). Additionally, systemic inflammation was measured by testing the serum for CRP on days 2 and 8 following the injection of the various treatments. No significant differences were found between the treatment groups in CRP values on either of the days tested (Figure 3G). H&E slides 2 days following injections were scored by a blinded pathologist for peripheral mononuclear cells, lymphocytes, macrophages and fibrosis, to reveal no differences between any of the groups nor within any inflammatory cell type (Figure 3H).

### MCM-LPX-FLuc and FMCM-LPX-FLuc do not inhibit fracture healing

**FIGURE 4:**
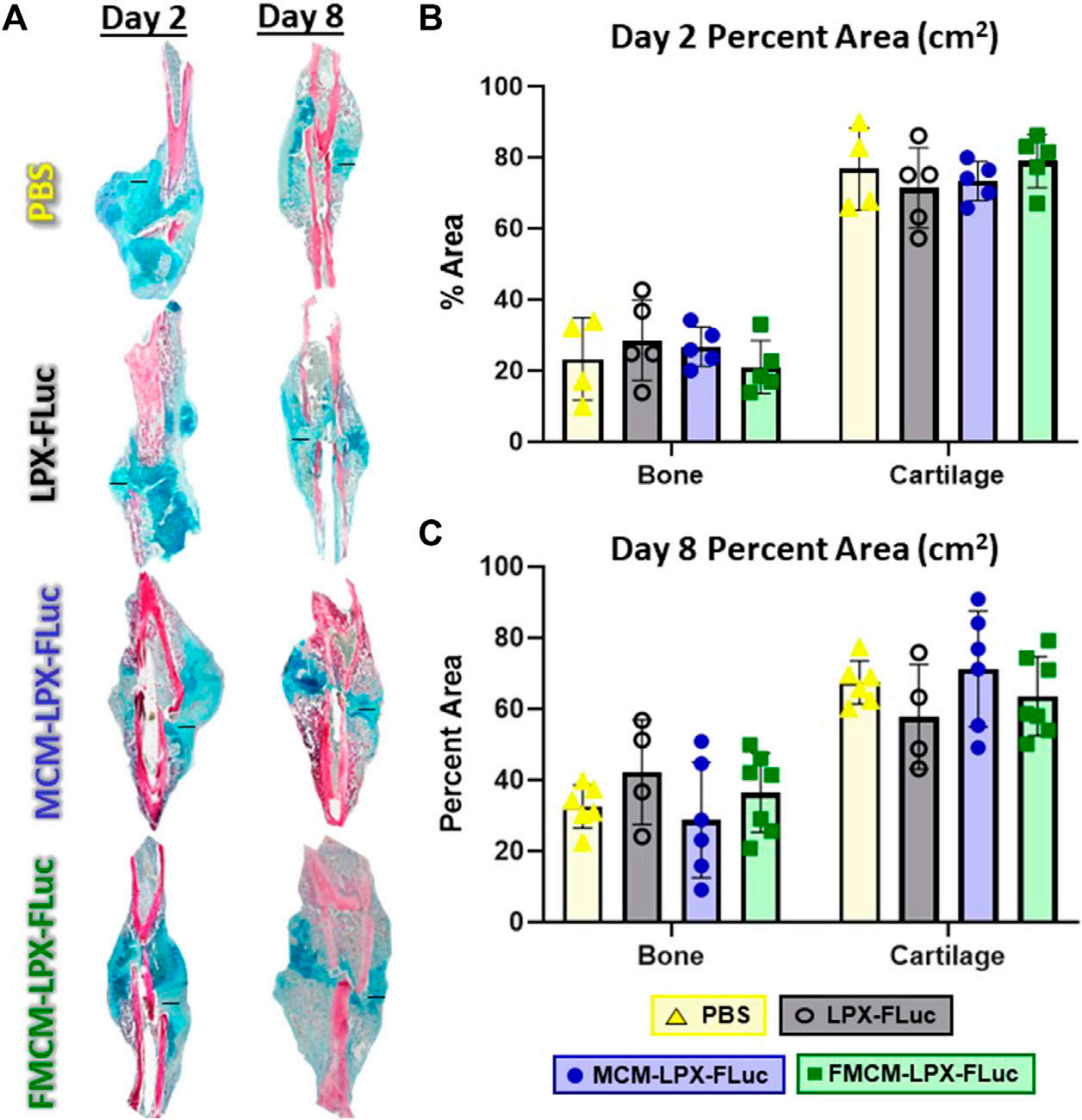
Fractured limbs were harvested, processed for histological analyses and stained with Hall Brundt’s Quadruple (HBQ) stain, where bone is stained red and cartilage is stained blue, scale bars represent 500 μm **(A)**. Histomorphometric principles were used to quantify tissue composition both 2 days after treatment **(B)** and 8 days after treatment **(C)**.

To test whether MCM-LPX-FLuc or FMCM-LPX-FLuc interfered with bone healing when delivered locally following fracture and stabilization, histomorphometry was performed 2 and 8 days following treatments. No significant differences were found between any of the groups tested in bone or cartilage tissue composition within the fracture callus at day 2 (Figure 4B) or day 8 (Figure 4C) following treatment.

### FMCM-LPX-FLuc prolongs transfection kinetics in a murine tibia fracture model

**FIGURE 5:**
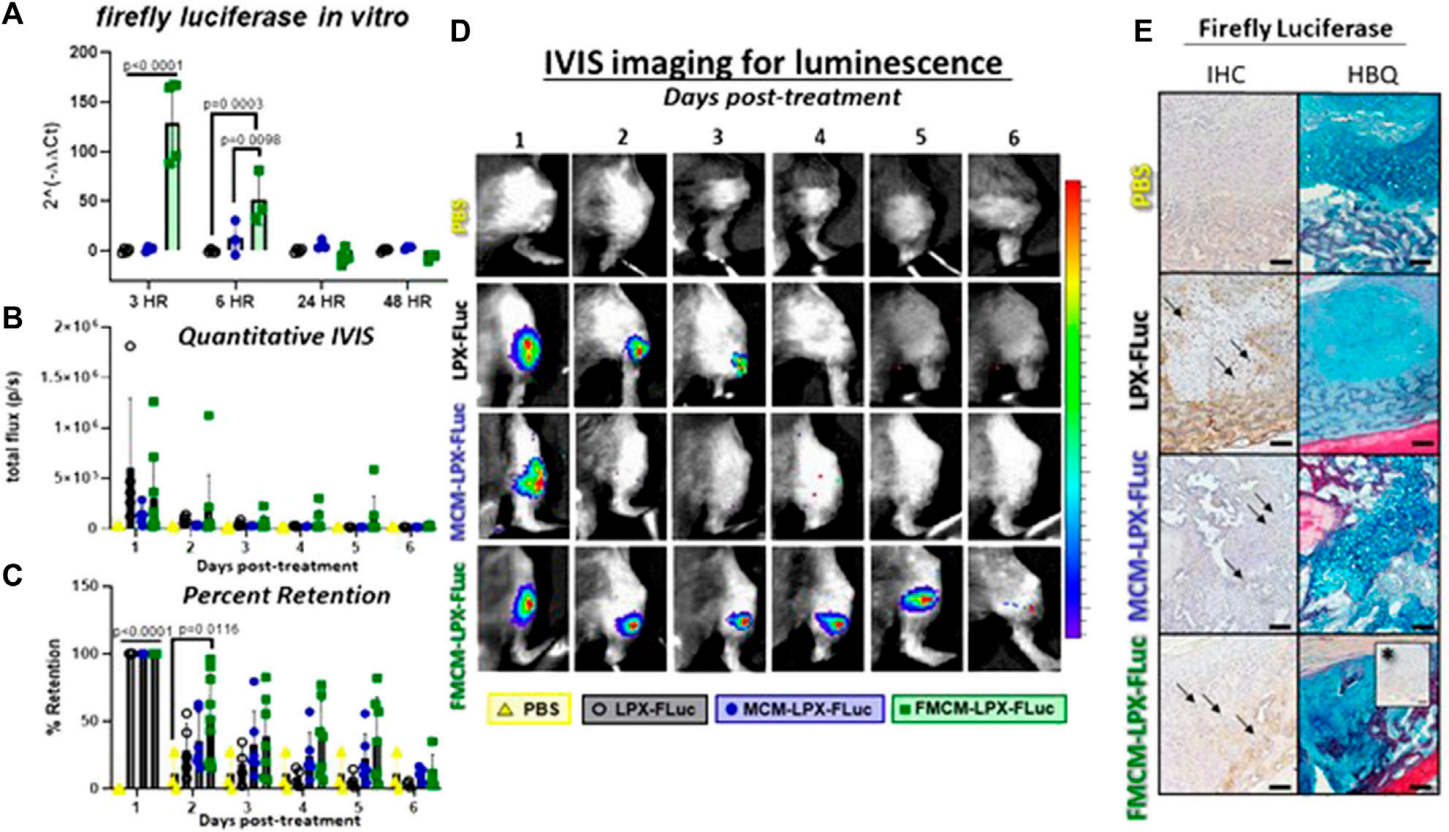
Transfection efficacy and kinetics were tested using firefly luciferase as a reporter gene to track and measure transfection. *In vitro* analysis was first measured in chondrocytes to determine transfection efficiency by testing firefly luciferase gene expression **(A)**. IVIS imaging was used for *in vivo* analysis to quantify luciferase signal and view expression **(B, D)**. Percent of luciferase signal retained within each mouse was measured for each treatment group **(C)**. IHC for firefly luciferase was used to qualitatively assess cells expressing the FLuc mRNA 2 days after local injections using adjacent slides stained with HBQ to determine cell lineage **(E)**. Arrows represent examples of positively stained cells for firefly luciferase protein. *Represents IgG negative control. Scale bars represent 100 μm.

MCM-LPX-FLuc and FMCM-LPX-FLuc were tested for their capacity to regulate lipoplex encapsulation and release dynamics. FMCM-LPX-FLuc was found to significantly enhance transfection to chondrocytes *in vitro* at 3 and 6 h following treatment compared to both LPX-FLuc and MCM-LPX-FLuc groups (Figure 5A). IVIS images show representative luciferase expression over time within each group (Figure 5D) along with the corresponding quantification of the regions of interest within each individual animal (Figure 5B). Interestingly, while FMCM-LPX-FLuc had the highest amount of luciferase expression *in vitro*, LPX-FLuc (*n = 5*) had the highest luciferase signal on the first day following treatment *in vivo*. In calculating the percent of luciferase signal retained within each mouse, FMCM-LPX-FLuc maintained significantly more luciferase signal over the PBS control 2 days after injections, when all other treatment groups lost significance (Figure 5C). The MCM-LPX-FLuc group showed an average sustained signal of only 1 day after treatment, with some mice in this group having no detected signal. For qualitative assessment, immunohistochemistry was performed for firefly luciferase 2 days after treatment to determine the cell type expressing FLuc mRNA (Figure 5E). The cell lineage was determined using adjacent slides stained with HBQ, revealing firefly luciferase expression within cartilage, and specifically within hypertrophic chondrocytes, and mesenchyme tissues. Arrows show examples of cells positive for expressing firefly luciferase.

## Discussion

Limited treatment options are available to facilitate fracture healing in non-union or delayed union cases. Most current treatments require additional surgeries which are often associated with long recovery times and increased work absences. Protein-based therapeutics, including the only FDA-approved osteoanabolic recombinant BMP-2, are the traditional approach to promoting fracture healing. However, proteins are typically delivered using supraphysiological doses as recombinant proteins are frequently challenged with low physiologic activity (Shields et al., 2006; Mumcuoglu et al., 2017; Khalil et al., 2020). Specifically, delivering high doses of recombinant BMP-2 results in high complication rates ranging from 20%–70% of all cases with complications ranging from mild to severe and potentially catastrophic (Shields et al., 2006; James et al., 2016). As an alternative, recombinant BMP-7 with collagen carrier was FDA approved in the past for impaired fracture healing cases, yet has since been removed from market (White et al., 2007; Gillman and Jayasuriya, 2021).

As an alternative to traditional protein based therapeutics, gene therapy proves to be an emerging field in bone regeneration with studies investigating genes encoding growth factors, transcription factors, and hormones involved in osteogenesis (De la Vega et al., 2021; Evans et al., 2021; Watson-Levings et al., 2022). De la Vega et al., 2021 has thoroughly reviewed preclinical studies which delivered BMP-2 cDNA using vector systems, like adenovirus and lentivirus. It was found that use of viral platforms to deliver gene encoding BMP-2 provoked an immune response and consequently impeded bone healing when tested *in vivo* (Lieberman et al., 1998; Musgrave et al., 1999; Egermann et al., 2006; Müller et al., 2017; Bougioukli et al., 2019). New strategies in gene therapy for bone regeneration involve use of mRNA, yet this approach is still challenged with short-half life and immunogenicity (Balmayor, 2022; Balmayor, 2023). To combat these challenges, other approaches have focused on modifying the mRNA sequence to maximize transfection while minimizing an immune reaction (Zhang et al., 2019; Balmayor, 2022). Despite these modifications, when testing chemically modified BMP-2 mRNA in various models, the use of liposomes in addition with a collagen sponge have frequently been employed as a delivery mechanism (Geng et al., 2021; De La Vega et al., 2022). Thus, it is imperative to develop biomaterials for mRNA therapeutics that facilitate an injectable application with effective transfection of cells, while minimizing immunogenicity.

In this study, we used a mineral coated microparticle (MCM) based platform to deliver mRNA encapsulated liposomes to a fracture site to evaluate their ability to effectively prolong mRNA delivery without promoting inflammation or inhibiting bone healing. Protein expression following transient transfection from mRNA is sustained for only a limited time, making it an ideal platform for bone repair, as stable transfection is more suited for chronic or genetic diseases (Agholme et al., 2010). Further, this transient gene expression necessitates the enhancement of mRNA stability from endonuclease-mediated decay (DR and Schoenberg, 2011). Until recently, the use of mRNA therapeutic platforms have been limited due to challenges associated with mRNA stability, cytotoxicity of the delivery platform, and induction of innate inflammation (Mockey et al., 2006; Zohra et al., 2007; Ramunas et al., 2015; Sultana et al., 2017). Cationic lipid vesicles such as Lipofectamine™, are frequently used *in vitro* to enhance mRNA stability, yet the cytotoxicity associated with this reagent limits clinical translation (Guo et al., 2019; Inglut et al., 2020). MCMs are comprised of calcium phosphate and biomimetic fluids, creating a mineral coating originally designed for controlled and localized delivery of growth factors (Choi and Murphy, 2010; Dang et al., 2016; Khalil et al., 2017). Recently, MCM lipoplexes have been utilized to enhance mRNA and cDNA stability, resulting in an increased transfection efficiency, enhanced cell internalization, and reduced cytotoxicity seen from cationic lipid vectors (Fontana et al., 2019). Additionally, it has previously been shown that mineral coatings display an osteogenic potential, but also can enhance transfection of plasmid DNA in bone marrow stem cells (Choi and Murphy, 2010; Choi et al., 2013).

Building on previous engineering efforts to develop the MCM platform for gene delivery, this platform was tuned to further advance mRNA delivery by the inclusion of a chemical dopant. Incorporating fluoride in the mineral coating has been shown to decelerate mineral dissolution which subsequently affected the protein binding and release kinetics for BMP2 (Yu and Murphy, 2014; Khalil et al., 2017). Fluoride has also been associated with activation of the canonical Wnt pathway, a critical pathway in bone formation (Guo et al., 2011; Pan et al., 2014; Nelson et al., 2022). Specifically, fluoride prevents the destruction complex from sending β-catenin to be proteolytically degraded, thereby increasing the nuclear localization of β-catenin (Pan et al., 2014). The transcription factor β-catenin modulates the expression of bone-associated genes, specifically *runt-related transcription factor 2* (*Runx2*) and *Osterix* (*Osx*) (Zhang et al., 2008; Cai et al., 2016). Importantly, while fluoride has frequently been tested as a treatment for osteoporosis and to enhance osseointegration, minimal efforts have focused on fluoride’s efficacy in fracture repair (SAVCHUCK, 1957; de Gubareff and Platt, 1969; Shteyer et al., 1977).

In this study, we evaluated the effects of FMCM-LPX on canonical Wnt activation and further osteogenic potential by probing for bone-related genes *Axin2*, *Runx2*, *Osx* and *Ocn* following treatment. While FMCM-LPX significantly activated the canonical Wnt pathway in chondrocytes *in vitro*, there was no significant upregulation of Wnt *in vivo*. While the same increased osteogenic effect was not observed in differentiated osteoblasts as in the progenitor chondrocytes, this may be due to the FMCM-LPX platform having more influence on cells in a less differentiated state, like progenitor cells. Multiple cell lineages comprise a fracture callus, all with varied states of differentiation, including both progenitor chondrocytes and osteoblasts. The *in vivo* gene expression data trended higher in osteogenic markers yet did not reach significance, suggesting more resemblance to that of the less differentiated cell type, progenitor chondrocytes. Additionally, the higher amount of cartilage composition, as evidenced through histomorphometry, confirms that more progenitor chondrocytes reside within the fracture as compared to osteoblasts at this time point. Thus, this heterogeneous cell population within a fracture callus may have hindered the osteogenic gene expression from reaching statistical significance. While fluoride has been heavily implicated in therapies that enhance bone mass, an adverse and deleterious effect at high doses (>8 mg/day) has been observed with chronic exposure (Grynpas et al., 2000; Du et al., 2022). Thus, only moderate amounts of FMCM-LPX-FLuc were tested *in vitro,* showing no cytotoxic effect on metabolic activity at any of the concentrations tested. Additionally, the concentration chosen for *in vivo* studies was previously used in a murine spinal cord injury model and was shown to not only effectively deliver mRNA, but also improve motor function (Khalil et al., 2022).

Since viral platforms delivering BMP-2 gene have been found to impede fracture healing due to an immunogenic response, it was important to determine whether immunogenicity was found using non-viral platforms (Egermann et al., 2006; De la Vega et al., 2021). Robust testing of localized and systemic inflammation revealed that delivery of FMCM-LPX-FLuc did not provoke a significant immunogenic response at either level. While this group did have the least pro-inflammatory effect *in vitro*, no significant differences were found when testing pro- or anti-inflammatory cytokines within the fracture callus. Interestingly, De La Vega et al., 2022 reported elevated levels of both pro-and anti-inflammatory cytokines in animals treated with synthetic RNA, yet were not elevated in animals treated with sham or protein groups. In this study, robust testing of localized and systemic inflammation revealed no acute inflammatory response found even 2 days after treatment with FMCM-LPX-FLuc or any treatment tested. This lack of immunogenicity may be due in part to a modest amount of mRNA being delivered (10 μg) in comparison to other reports (50 μg) (De La Vega et al., 2022).

This study had several limitations including use of Lipofectamine™ at various times within the shelf-life range, which impacts the transfection potency of cells as the reagent diminishes in potency with increased time on the shelf. To mitigate this variance, all experiments were executed using the same Lipofectamine™ vial so that all treatment groups were comparable. Additionally, only one of the concentrations of MCM-LPX-FLuc and FMCM-LPX-FLuc was tested *in vivo* for enhanced osteogenic markers and for stimulation of bone. Since there was a minimal osteogenic response using FMCM-LPX-FLuc, future work will include testing a functional mRNA sequence involved in promoting bone, such as BMP-2 or BMP-7 (Gao et al., 2014; Gao et al., 2022). One last limitation of the study includes the lack of biodistribution tested following treatment. While a localized delivery may only minimize biodistribution of the mRNA lipoplexes, this should be further examined through analysis of main organs including liver, lung, spleen, kidney and heart tissues.

The mRNA delivery platform developed in this study, FMCM-LPX-FLuc, was found to prolong the transfection kinetics of luciferase, without provoking immunogenicity nor interfering with fracture repair. This data proves to be consistent with prior data showing fluoride dopant to decrease mineral dissolution in MCM and to prolong delivery of growth factors and nucleic acids (Khalil et al., 2017; Fontana et al., 2019). In this manuscript, we explored the safety and efficacy of this novel mRNA delivery platform prior to administering a therapeutic gene. The decision to utilize the luciferase reporter system was based on its well-established detection methods, which enable accurate and non-invasive monitoring of transfection efficiency and kinetics. Furthermore, it provided us with the means to confirm the absence of any cytotoxic or adverse immune reactions, which are crucial factors to consider prior to introducing osteoinductive mRNAs, especially in a fracture healing application. We think that the osteogenic potential of this platform could be maximized through addition of a functional mRNA transcript like BMP-2, or BMP-7.

## Scope

The scope of this manuscript is to test biomimetic mineral coated microparticles (MCM) as an mRNA delivery platform for fracture healing applications. MCM and additionally a fluoride-doped MCM (FMCM) were tested in its osteogenic potential and capacity to provoke a robust inflammatory response in a murine fracture healing model. Bone formation was then quantified histologically and finally, reporter gene, Firefly Luciferase, was used to track and measure transfection *in vitro* and *in vivo*. The scope of this work specifically tests an advanced biomaterial for the regeneration of bone and is well suited for Research Topic: Advanced Biomaterials for Hard Tissue Repair and Regeneration.

## Data Availability

The original contributions presented in the study are included in the article/Supplementary Material, further inquiries can be directed to the corresponding authors.
