# Supplementary material for: Mineral coated microparticles doped with fluoride and complexed with mRNA prolong transfection in fracture healing
Source: Front Bioeng Biotechnol. 2024 Jan 9;11:1295313. doi: 10.3389/fbioe.2023.1295313 (PMC10803474; doi:10.3389/fbioe.2023.1295313)
Supplement: Supplementary file 1 [file Table1.DOCX]

**Supplemental Figures**


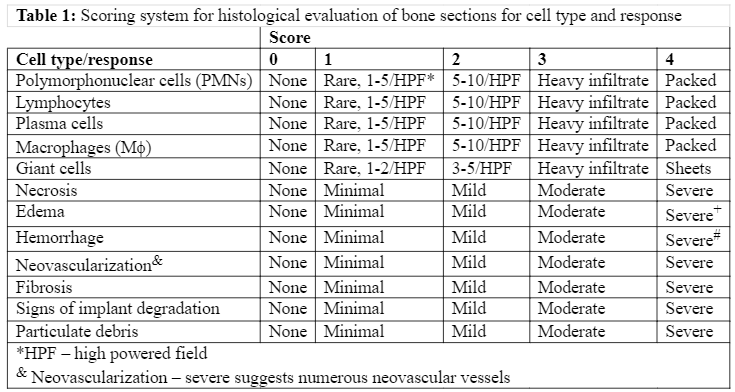


**Supplemental Table 1.** Histopathology results used a Semi-quantitative scoring method using a modification of ISO10993-6 Annex E: Biological evaluation of medical devices – Part 6: Tests for local effects after implantation.
